# Empyema of the Left Plural Cavity Treated by Injection of Iodine

**Published:** 1857-12

**Authors:** 


*Case of Empyema of the left plural cavity treated by injection of iodine.* By DANIEL BRAINARD, M. D., Professor of Surgery in Rush Medical College, etc.; reported by Edwin Powell, M. D.

Thomas S. Bryan, aged 16, a native of Norway, first came under the care of Prof. Brainard, April 15, 1857, with empyema of the left plural cavity, the result of a wound.

The previous history of the case, as given by his friends, is as follows: Some five months since the patient received a stab in the left side, between the fifth and sixth ribs, just below the nipple, with a sharp-pointed knife, which was followed by difficulty of breathing, spitting of blood, etc. The external wound was closed with sutures and adhesive plaster, the patient was bled, purged and confined to a low diet. At the end of three weeks the wound had healed, and the patient was able to be about, but the difficulty of breathing had not entirely subsided, and he suffered more or less pain in that side.

He remained in this state for the space of two months, when a pretty copious discharge of matter took place from the wound, which had opened by ulceration. Since which time there has been a constant discharge of extremely fœtid pus from the wound, and the patient was gradually becoming weaker and emaciated. The parents of the patient being in destitute circumstances, nothing was done for him during this time.

*Present Condition.*—The patient is now excessively thin; the skin presents a shrivelled and yellow appearance. There is a distinct bulging of the affected side, and one teacupful of fetid pus is discharged each day from the wound. The shoulder of the affected side is two inches lower than the other. He walks with the utmost caution, as the least jar or mis-step gives him great pain; in fact, he is scarcely able to walk from one room to another without assistance. He is also affected with loss of appetite, chills, night sweats and diarrhœa.

*Treatment.*—The opening was sufficiently enlarged to admit a gum elastic tube of four inches in length, of the size of a No. 9 catheter, through which the pleural sac was washed out, morning and evening, with the following solution:

℞ Iodinium, j gr.
Potassi Iodidum, iij grs.
Aqua Destillata, ℥i.
M. Ft. Sol.

The solution was thrown in with a strong syringe, and allowed to flow out again after remaining in for a few moments. If any smarting followed the operation, the cavity was washed out with cold water. The first effect of the iodine was to change the character of the discharge to nearly healthy pus, and afterwards to lessen the amount of suppuration. No bad symptoms at any time followed the use of the injection. Iodine could be detected in the urine. The patient was, in the meantime, placed upon as generous a diet as he could take, with the use of stimulants.

As he had a very intelligent friend to take charge of him, he was allowed to go Into the country to his own home, and continue this course of treatment there, after having remained under observation for two weeks.

*July* 10*th*.—The patient returned to the office this morning from the country. It was surprising to see the improvement that had taken place; he has gained in flesh and strength quite rapidly. Suppuration is still going on, but not much, and the discharge is of a healthy character. The injections had been faithfully used while in the country. He was advised to pursue the same plan, and again returned to his home.

*October* 10*th*.—The patient again presented himself at the office, from the country. The suppuration had entirely ceased, and the external wound had healed soundly. Instead of the mere skeleton he was seven months ago, he now presents the appearance of a sound healthy boy.

I made a physical examination of his chest, this morning, and could detect no difference in the two sides.

I am not aware that injections of iodine have ever been used or recommended in this coun:ry in cases of empyema, heretofore, but such beneficial results followed their use in this case, that I think we should be justified in resorting to them in similar cases.—*North-Western Med. and Surg. Jour.*

---

*Transmission of the Virus of Grease from the Horse to Man.*—Drs. Maunoury and Pichot have published an interesting series of experiments, tending to prove the identity of grease and cowpox. This doctrine, which was always maintained by Jenner, has received confirmation from the observations of Loy, Godine and others.

The following is a summary of the facts related by Drs. Maunoury and Pichot. François Barthélemy B——, aged twenty-eight, of lymphatic temperament, a farrier, presented himself to Dr. Pichot on the 5th of March, 1856. He had not been vaccinated. On the backs of his hands, which were red and swollen, were several confluent opaline pustules, depressed in their centre, having all the appearance of vaccine pustules of the eighth or ninth day. The inflamation, with which the pustules were surrounded, had appeared on the second, the pustules themselves preceded the inflamation some days. This man had not been in contact with any cow, but on the 11th of February he had shod a horse suffering from grease. There existed, at the time, numerous cracks about his hands. The disease from which the horse was suffering was certified by a qualified veterinarian. Various inoculations were practised with the liquid taken from the pustules presented by B——, with the effect of reproducing the same disease. The most perfect set of experiments were made by M. Maunoury, who transmitted the virus through four sets of cases. The following are the results of his observations:—

1. That virus obtained from the hands of the farrier B——, and innoculated on the arm of an infant, produced a full pustule, having all the characters of a vaccine pustule—form, evolution, termination.
2. That lymph taken from this pustule, and inoculated on the arms of three persons, had produced identical pustules, which are truly vaccine.
3. That the transmissicn of the virus by successive generations, has not diminished the intensity of the force of the poison. One of the set of cases presented large pustules, depressed in the centre, and filled with matter; each pustule served for several inoculations, and the charging of several sets of glasses.

4. That from these facts it is evident that the virus taken from the pustules of the farrier, was identical with the vaccine.—*Archives Génerales de Médecine*, April, 1857, pp. 365, 398, from *the Brit. & For. Med. Chi. Rev.*

---

*Extirpation of the Supra-Renal Capsules, Spleen and Thyroid Gland, from living Animals.*—M. Philipeaux addressed a note to the Academy of Sciences, in which he says that he has succeeded, not only in removing the supra-renal capsules, but also the spleen and thyroid body in two white rats (Mus Rattus,) aged one month. By an operation of the kind described in my first note, I have removed first the right supra-renal capsule, and in ten minutes afterwards the left. These animals in about one month were completely recovered, when I proceeded to remove the spleen through a small opening in the side of the abdomen, after which the wound was closed by a single suture. It was but a short time until they had again completely recovered, and I was successful in removing from the same animals the thyroid gland through a longitudinal section in the anterior part of the neck, on a level with the tracheal artery. At this time these animals, aged three months, are in excellent health, notwithstanding they have been despoiled of their supra-rena capsules for sixty-seven, the spleen for twenty-six, and the thyroid body seven days. I take this opportunity of informing the Academy also, that I possess one male rat, living in fine condition, which has been for four month deprived of his supra-renal capsules, and a female for forty-three days, without any modification of their functions that can be perceived. The female has been impregnated, borne eight young ones, and is raising them.

These experiments prove still more thoroughly the truth of my former conclusions, (viz., that animals can live without the supra-renal capsule.—Trans.) I adjoin to these, that they can live after being deprived at the same time of the supra-renal capsule, the spleen and the thyroid body, and that consequently none of these (non essential bodies) are required to, or can supplementarily discharge the function of the other.—*Gaz. des Hos.*, from *The American Med. Gaz.*

---

*Congenital Absence of the Supra-Renal Capsules.*—M. Martone addressed a description, accompanied with a drawing, of a malformation which came under his observation, in which there was a fusion of both kidneys, they forming but one body, with a congenital absence of the supra-renal capsules.—*Gaz. des Hos.*, from *The American Med. Gaz.*

*Symptoms of Poisoning produced by Ergot,*—The following case occurred to M. Tiastour: A woman of lymphatic temperament, and very fat, had profuse uterine hæmorrhage after delivery of a still-born fœtus at eight months. Three *grammes* (forty-five grains) of ergot were given in six doses in the course of an hour; cold applications were also made to the abdomen and thighs. The hæmorrhage was checked and the uterus contracted, but in a few hours there were severe symptoms of disturbance of the circulation. The patient rolled constantly from one side to the other; her face was pale, her lips a little blue. The pulse in the radial and brachial arteries was imperceptible; the heart-beat was regular, not increased in frequency, but weak. The patient complained of prickling sensations, and of cold in her hands and feet; she said that she no longer felt her limbs. The extremities were really cold to the touch; the skin and nails had a blue tint, both in the hands and feet; intellect was clear. These symptoms were combated by the alternate administration of broth and wine, then by some spoonfuls of opiate syrup given every two hours. All the alarming symptoms had disappeared in three days; convalescence was tedious, but perfect recovery took place.—*Journal de la Section de Médecine de la Loire-Inférieure,* and *Gazette Médicale de Paris,* July 25th, 1857, from *the Brit. and For. Med. Chi. Rev.*

---

*Salt not a Necessary of Life.*—That salt performs an important office in sanguification, and is regarded by physiologists as a necessary article in our dietary, is well known. It is therefore in use among all nations, from the savage to the most civilized and enlightened people. There are, however, a few members of the whole human race to whom the use of table salt is unknown, among whom may be named the inhabitants of Mauritius and the Patagonian Pampas of America (Faerichs). I have, says M. Gatton, reeently learned of another tribe who never eat salt. It is the Damaras of tropical South Africa. In their country there is no salt. In Europe it is universally believed that salt is indispensable to life, but M. Gatton in the countrv of Damaras saw this proven to be a fallacy. He made a journey with eleven other men on horse-back of six weeks' duration, with only a pill-box full of salt. The whole of them used no more than this. They eat nothing but flesh, and drank coffee alone. There can be no doubt that people who live on flesh and milk need much less salt than those whose sole nourishment is derived from the vegetable kingdom. The half of the people of Damara eat nothiug but pigg-nuts, the most meagre and indigestible of nourishment, and which must be taken in very large quantities to afford subsistence enough. The Hottentots of Wallfish Bay, who live aimost entirely on squashes, with the sea on one side and salt springs on the other, take no pains to collect salt for use, which they certainly would do if they experienced the same need of it as the Europeans. Wild animals in Swakop, according to Gatton, do not frequent salt licks like they do in America. He visited these places, and although the tracks of wild animals were abundant, they were a month old, and indicated that they were passing without coming near enough the salt rocks to lick them. He mentions tribes also that not only eat their food without salt, but actually loathe it.—*Allge. Med. Cent. Zeitung,* from *The American Med. Gaz.*

